# Do Area-Level Environmental Factors Influence Employment for People with Disability? A Scoping Review

**DOI:** 10.3390/ijerph19159082

**Published:** 2022-07-26

**Authors:** Nicola Fortune, Bernadette Curryer, Hannah Badland, Jennifer Smith-Merry, Alexandra Devine, Roger J. Stancliffe, Eric Emerson, Gwynnyth Llewellyn

**Affiliations:** 1 Centre for Disability Research and Policy, The University of Sydney, Camperdown, NSW 2006, Australia; bernadettecurryer@gmail.com (B.C.); jennifer.smith-merry@sydney.edu.au (J.S.-M.); roger.stancliffe@sydney.edu.au (R.J.S.); gwynnyth.llewellyn@sydney.edu.au (G.L.); 2 NHMRC Centre of Research Excellence in Disability and Health, Parkville, VIC 3010, Australia; hannah.badland@rmit.edu.au (H.B.); alexandra.devine@unimelb.edu.au (A.D.); eric.emerson@lancaster.ac.uk (E.E.); 3 Centre for Urban Research, RMIT University, Melbourne, VIC 3000, Australia; 4 Centre for Health Equity, Melbourne School of Population and Global Health, University of Melbourne, Melbourne, VIC 3010, Australia; 5 Centre for Disability Research, Faculty of Health and Medicine, Lancaster University, Lancaster LA1 4YW, UK

**Keywords:** employment, disability, environmental factors, area-level, geographic, inequalities, labour force

## Abstract

Employment is an important social determinant of health and wellbeing. People with disability experience labour market disadvantage and have low labour force participation rates, high unemployment rates, and poor work conditions. Environmental factors are crucial as facilitators of or barriers to participation for people with disability. Understanding how the physical, social, and economic characteristics of local areas influence employment for people with disability can potentially inform interventions to reduce employment inequalities. We conducted a scoping review of research investigating associations between area-level environmental factors and employment for people with disability. Eighteen articles published between 2000 and 2020 met the inclusion criteria, and data were extracted to map the current evidence. Area-level factors were categorised into six domains relating to different aspects of environmental context: socioeconomic environment, services, physical environment, social environment, governance, and urbanicity. The urbanicity and socioeconomic environment domains were the most frequently represented (15 and 8 studies, respectively). The studies were heterogeneous in terms of methods and data sources, scale and type of geographic units used for analysis, disability study population, and examined employment outcomes. We conclude that the current evidence base is insufficient to inform the design of interventions. Priorities for future research are identified, which include further theorising the mechanisms by which area-level factors may influence employment outcomes, quantifying the contribution of specific factors, and interrogating specific factors underlying the association between urbanicity and employment outcomes for people with disability.

## 1. Introduction

People with disability have a right to work on an equal basis with others. As stated in Article 27 of the United Nations Convention on the Rights of Persons with Disabilities (CRPD), “this includes the right to the opportunity to gain a living by work freely chosen or accepted in a labour market and work environment that is open, inclusive and accessible to persons with disabilities” [1]. However, globally, people with disability experience disadvantage in the labour market [2].

Compared with their nondisabled peers, people with disability have lower rates of labour force participation and higher rates of unemployment [3,4]. Further, those with a job are more likely to be employed under nonstandard conditions and to experience disadvantage in relation to job security, retention, satisfaction, earnings, and characteristics related to psychosocial job quality, such as job control and level of job demands [5,6,7,8,9,10,11]. Employment is a well-known social determinant of health, with unemployment, underemployment, job insecurity, and nonstandard conditions of employment detrimentally affecting health and wellbeing [12,13,14]. For people with disability, there is evidence that adverse employment outcomes (such as unemployment and underemployment) negatively impact mental health, and that such impacts can be greater than those for people without disability [15,16].

The importance of equal access to *decent work* is recognised in Goal 8 of the United Nations 2030 Sustainable Development Agenda [6,17]. Not having a job or having a job that negatively impacts the individual (such as poor work conditions, poor remuneration or job insecurity) can compound disability-related disadvantage. The link between disability and poverty is well established [18,19,20]. As stated in the *World Report on Disability*, “If people with disabilities and their households are to overcome exclusion, they must have access to work or livelihoods, breaking some of the circular links between disability and poverty” [2] (p. 236).

### 1.1. Area-Level Environmental Factors

The International Classification of Functioning, Disability, and Health (ICF) recognises the crucial role of environmental factors as facilitators of or barriers to activities and participation for people with disability [21]. Environmental factors “make up the physical, social and attitudinal environment in which people live and conduct their lives” [21], and can operate at the individual (micro), community (meso), and societal (macro) levels [22].

There is an established body of research on the influence of the places where people live on health, wellbeing, life opportunities, and outcomes [23,24]. In particular, neighbourhood effects research interrogates “the idea that living in more disadvantaged neighbourhoods has a negative effect on residents’ life chances over and above the effect of their individual characteristics” [25] (p. 2787). Informed by this research, place-based interventions seek to change physical, social, or economic factors to improve outcomes for the people who live within geographically defined communities [26,27,28]. There is a growing literature concerning the influence of environmental factors on social and economic participation for people with disability [29,30,31,32,33]. However, little research has looked at how the characteristics of the local areas in which people with disability live may operate as barriers to or facilitators of participation.

Area-level factors that could be expected to impact employment for people with disability include: local infrastructure that affects access to employment, such as street conditions, public transport, and the accessibility of public buildings [34,35]; and social and economic characteristics, such as community social capital, local networks, neighbourhood safety, local unemployment, and local job availability. For example, some residential locations may offer poorer access to jobs due to distance or limited transport options, whereas in some locations, strong labour market networks among residents may enable individuals to connect more readily with potential employers [36,37,38].

Government policies that aim to increase employment rates for people with disability utilise mechanisms such as structuring income support payments to encourage people with disability into the labour market, proscribing employment discrimination through legislation, incentivising and supporting employers to hire and retain workers with disability, and supporting individuals to obtain and maintain employment [39,40]. Little attention has been paid to disability employment policy and programming regarding the influence that geographic location can have in shaping employment opportunities for people with disability. Understanding how area-level environmental factors influence employment outcomes for people with disability could inform more effective policies and programmes to reduce inequalities. For instance, factors such as accessible public transport or employer attitudes towards workers with disability might be identified as levers for intervention within geographically defined localities. Additionally, employment support services delivered to individuals could be tailored to take account of the characteristics of local areas. To inform potential interventions, evidence is needed about associations between area-level factors and employment for people with disability, including how, how much, and in which circumstances specific factors influence employment outcomes.

### 1.2. Objective of This Scoping Review

To our knowledge, no published review has examined the evidence on how area-level environmental factors influence employment outcomes for people with disability. To address this gap, our objective was to identify published empirical research that has investigated associations between area-level factors and employment for people with disability. Drawing this evidence together is valuable for informing policy and practice, and developing future research agendas.

## 2. Materials and Methods

Scoping review methodology was selected because our purpose was to describe the nature and extent of the available evidence, not to summarise research findings. Scoping reviews are commonly conducted to map key concepts, clarify conceptual boundaries, describe the types of available evidence, and identify knowledge gaps [41,42]. Because scoping reviews aim to provide an overview of available evidence, the methodological quality or risk of bias of included studies is generally not assessed [43].

We used a systematic method to identify studies and extract relevant information, drawing on the methodological framework identified by Arksey and O’Malley [41], and refined by Levac and colleagues [44]. This framework consists of five key stages: identifying the research question; identifying relevant studies; study selection; charting the data; and collating, summarising, and reporting the results.

### 2.1. Identifying the Research Question

Our research question was: what is the nature and extent of research examining associations between area-level environmental factors and employment for people with disability?

### 2.2. Identifying Relevant Studies

An online search of six databases, namely, Cinahl, Embase, Informit, Medline, Scopus and Web of Science, was undertaken. The search strategy was developed by NF and BC with advice from an academic librarian. The following search terms were used:

1. Employ* OR unemploy* OR job* OR labo*r force OR workplace;
2. disabilit* OR disable* OR impairment*;
3. local area OR social environment* OR physical environment* OR geograph* OR contextual factor* OR built environment* OR neighbo*rhood*

These three sets of terms were joined by ‘AND’. Results were filtered for peer-reviewed journal articles written in English and published between January 2000 and September 2020. January 2000 was chosen as the start date to capture the body of research produced over two decades, a time period that included the publication of the ICF in 2001, bringing a new focus to environmental factors in disability research, and the adoption of the CRPD in 2006. The end date of 2020 was determined by the available funding for this research.

### 2.3. Study Selection

Inclusion criteria were identified and refined by the review team according to the schema set out in the JBI Manual for Evidence Synthesis [43]:

*Population*: People with disability, defined as having a long-term impairment, activity limitation, or participation restriction associated with a health condition (including episodic conditions such as mental illness). We included studies in which people with disability had been identified on the basis of accessing disability-specific services or programmes (e.g., vocational rehabilitation, disability income support payments).

*Concepts*: Area-level factors associated with employment outcomes. We operationally defined area-level factors as aspects of the physical, social, economic, service, or policy environment specifically viewed from a spatial/geographic perspective (e.g., built environment features or unemployment rate within geographically defined areas). We broadly defined employment outcomes to include individual outcomes related to paid work, voluntary work or work experience (e.g., labour force participation, employment status, job conditions, pay, job satisfaction).

*Context*: Our focus was on studies investigating area-level factors operating in localities where people live at geographic scales that corresponded to people’s daily activities and working lives. We excluded studies using large geographic units such as state level in the USA or provincial level in Italy.

*Types of evidence sources*: Original empirical research including quantitative, qualitative, and mixed-methods study designs, published in English in a peer-reviewed journal.

The database search identified 4176 articles. After the removal of 2055 duplicates, 2121 articles were screened. Data management software Covidence (2020) was used to assist with screening. The title and abstract of each article were independently screened by BC and one other author (NF, GL, or JSM). Any disagreements were discussed by BC and NF until consensus had been reached. This process resulted in 133 articles selected for full-text review. An additional 26 articles were subsequently identified via a manual search of the references of the included articles and literature reviews identified by the original search. Following an independent title and abstract screening of these 26 articles by BC and NF, 8 articles were added for full-text review.

**Figure 1:**
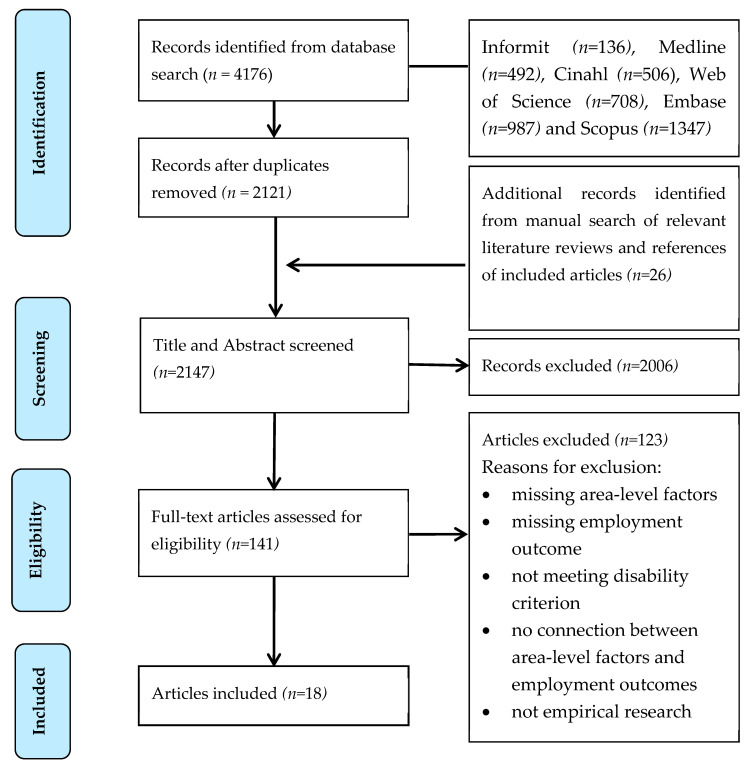
Literature search and screening PRISMA diagram.

The full text of the 141 articles was reviewed independently by BC and NF, followed by a discussion of differing views until consensus had been reached. This process resulted in 18 articles being selected for inclusion. The most common reasons for exclusion at the full-text review stage were area-level factors not being considered, and employment outcomes not being investigated. The search and screening process is summarised in Figure 1.

### 2.4. Charting the Data 

Following discussion among the review team, the key information for extraction was determined. Using Covidence (2020) software, we developed a template to guide data extraction. For the first five articles, data were extracted independently by NF and BC. The results were compared and discussed to ensure consistency and relevance to the research question. Data from the remaining articles were then extracted by either NF or BC.

### 2.5. Collating, Summarising, and Reporting the Results

We transferred the extracted data into a spreadsheet and produced a descriptive summary of each article including the author, year, purpose, and country of the study, study methods (including data sources and geographic units), the disability study population, employment outcomes, and examined area-level factors.

We categorised area-level factors using a framework of six domains relating to different aspects of environmental context. This framework was based on a conceptual model comprising five domains for classifying characteristics of neighbourhoods that influence early childhood development, which Goldfeld and colleagues developed drawing upon existing models and frameworks in the neighbourhood effects literature [45]. We added a sixth domain, urbanicity, as many of the studies that we identified focused on urban–rural differences. 

The six domains were:

**Socioeconomic environment**—covering sociodemographic factors such as population age structure, income, educational attainment, labour force status, and ethnic mix, and features of the local economy such as job availability, industry mix, and economic regeneration and development.

**Services**—provision of and access to services, both disability-specific services (e.g., disability employment services) and mainstream services (e.g., banks, shops, government-provided services); measures of service quality and distribution in relation to need.

**Physicalenvironment**—including roads, footpaths, parks, housing, presence and accessibility of public transport, and land use patterns.

**Social environment**—including social norms, community social capital, trust, crime, safety, social support networks, civic engagement, and neighbourhood attachment.

**Governance**—covering factors such a policies implemented at local level, leadership, governance structures, partnership structures, and decision-making forums.

**Urbanicity**—categorisation such as urban, suburban, rural, metropolitan, and nonmetropolitan, based on measures of population density, infrastructure, and/or distance to large cities.

## 3. Results

Table 1 presents a summary of the 18 articles listed alphabetically by the first author. In the Results Section, numbers in parentheses refer to the articles as numbered in Table 1.

### 3.1. Study Country and Year of Publication

Of the 18 articles, 13 were conducted in the USA. Other represented countries were Canada, Australia, the Netherlands, Sweden, and the UK, with one article each. No articles reported research undertaken in low- or middle-income countries. Six articles were published between 2000 and 2009, and 12 between 2010 and 2020.

### 3.2. Study Purpose

Fifteen studies had a specific and stated focus on understanding factors operating at the area level that affect employment outcomes for people with disability (2–13, 16–18). The remaining three studies examined area-level factors, although area-level factors were not mentioned in the study aims (1, 14, 15).

Ten studies (1, 4, 6, 8–13, 15) were related to vocational rehabilitation or supported employment programs, with many setting out to inform the more effective delivery of these services. Two studies examined how area-level factors interacted with government policies intended to increase employment rates for people with disability (5, 16).

### 3.3. Study Methods and Data Sources

We identified 15 quantitative, 1 qualitative (11), and 2 mixed-method studies (6, 7). Among quantitative studies, there was substantial variation in study design and methods, including randomised trial (4), spatial analysis (5), the analysis of linked data from two or more datasets (2, 15, 16), and the analysis of data from a single dataset (17, 18). Pre-existing data sources were used in most cases, including national statistical collections (2, 3, 13, 16–18), administrative data (5, 6, 8, 9, 15), and disease or disability registers (2, 7). Seven studies collected their own data (1, 4, 10–14). Several used a combination of different data sources. Only one study (15) used longitudinal data to examine temporal associations between area-level environmental factors and employment outcomes.

### 3.4. Geographic Unit

The nature and size of geographic units under investigation differed widely. The geographic unit often appeared to be dictated by the available data (e.g., data on unemployment rates at the county level). Geographic units included county (3, 5, 16, 17), municipality (5), service agency area (1, 6), commuting area (8,9), community (3), intraurban zones (17), and geographic units defined for national statistical purposes (*18*). Many studies that examined urbanicity did not specify the geographic unit of analysis (7, 10–14). For example, Hollick and colleagues [52] stated that postcodes were used to determine whether participants lived in rural or urban areas according to the 2011 UK Census, with rural areas defined as settlements of less than 3000 people.

### 3.5. Disability Study Population

The disability study population varied between studies. Seven considered disability in general (5, 8, 9, 11, 16–18). Nine focused on specific disability subgroups: psychiatric disability (1, 4, 6, 15), spinal cord injury (2), traumatic brain injury (10), axial spondyloarthritis (7), intellectual disability (14), and adolescents with severe disability (3). One study (13) excluded people with intellectual disability, and one (12) compared people with different levels of disability severity. Only one study compared people with and without disability (17).

### 3.6. Employment Outcomes Examined

Being in paid employment was the outcome most often examined (seven studies: 1, 2, 4, 6, 10, 12, 14). Other investigated outcomes were employment conditions (e.g., self-employed, earnings, hours/week, wages, commute time; 8, 9, 16, 17), access to or uptake of employment support services (10, 15), paid work experience during high school (3), industry type and work impairment (7), sick leave and unemployment duration (13), labour force participation rate (18), and spatial matching of quota jobs (i.e., job openings for disabled people made available in accordance with a mandatory quota system) to the target group of people with disability (5).

Disability was identified variously, for example, by virtue of being a vocational rehabilitation/supported employment client (4, 8–13), having a history of special education (3, 14), being identified as having a disability in population survey data (16, 17), being in receipt of a disability support benefit (5), or reporting a need for assistance with core activities (self-care, mobility, or communication) in a national census (18).

### 3.7. Investigated Area-Level Environmental Factors

We categorised the investigated area-level factors into the six domains described in Section 2.5. Results are presented in Table 2. Most studies investigated area-level factors in one or two domains. Three studies investigated factors across three or more domains (1, 3, 16). No studies investigated factors across all six domains. In seven studies, urbanicity was the only represented area-level factor domain. The most commonly investigated area-level factors were in the domains of urbanicity (15 studies) and socioeconomic environment (8 studies). Not all aspects of each domain, as detailed in Section 2.5, were examined. For example, although the physical environment domain included roads, footpaths, parks, housing, the presence and accessibility of public transport, and land use patterns, only transport was investigated in studies identified in this review (1, 3, 16).

All the included studies found some associations between area-level factors and employment outcomes for people with disability. Our focus in this scoping review was to describe the nature and extent of the evidence base; therefore, we did not evaluate the strength of or attempt to summarise findings concerning associations between specific area-level factors and employment outcomes. Instead, we highlight some findings below as examples of the nature of the available evidence. Supplementary Table S1 sets out specific findings reported in each study, by domain.

#### 3.7.1. Urbanicity

Fifteen studies examined urbanicity, and seven of these did not examine factors in any other domain. In five studies, living in an urban setting was associated with more favourable employment outcomes for people with disability (2, 6, 7, 13, 14). Two found no association between rural or urban geography and employment outcomes (1, 3). Other studies reported more nuanced findings concerning urbanicity; for example, one found the likelihood of employment to be higher in rural than that in urban areas for people with severe disability, and the opposite association for people with non-severe disability (12). Other differences linked to level of urbanicity included job type (7), whether educational attainment was associated with employment outcome (12), wage gap between workers with and without disability (17), rates of vocational rehabilitation case closures to self-employment (8), and associated differences in hourly earnings and hours worked per week (9).

#### 3.7.2. Socioeconomic Environment

Measures of area-level employment or unemployment were included in all eight studies that investigated socioeconomic factors (1, 2, 4, 8, 13, 15, 16, 18). Two of these studies included additional variables measuring aspects of the local labour market—share of jobs in blue-collar industries (16) and employment market index, a constructed variable intended to capture the level of employment activity and opportunity (standardised ratio of number of employees to number of working-aged residents within an area) (18). In most cases, a positive association was found between favourable local labour market conditions and employment outcomes for people with disability, although there was no association in one study (2).

Other factors examined in this domain were area-level socioeconomic index, educational attainment, ethnic mix, the proportion of people who mainly speak English at home, poverty rate, household income, male-to-female sex ratio, and disability prevalence (2, 8, 16, 18). One study examined sociodemographic characteristics of the population with disability and the population without disability in relation to labour force participation of people with disability (18).

#### 3.7.3. Services

Four studies examined area-level factors related to service presence or accessibility: employment support services (6), school-based job skill programs (3), supported employment providers (15), and concentration of physicians (16).

#### 3.7.4. Governance

Two studies examined area-level governance factors. One reported no association between a measure of local government fiscal health and employment rates for people with disability (16). One reported a spatial mismatch between the availability of quota jobs and the location of the target group of people with disability (5).

#### 3.7.5. Physical Environment

Three studies investigated local availability of public transport (1, 3, 16), and one (3) also investigated the availability of accessible or disability-specific transport.

#### 3.7.6. Social Environment

Two studies examined area-level social factors: the proportion of residents who undertake voluntary work (18), and crime rate (16).

## 4. Discussion

The objective of this scoping review was to identify and describe the body of existing research investigating associations between area-level environmental factors and employment for people with disability. Below, we summarise the nature and extent of the evidence base, highlight some key gaps and limitations, and consider the implications of our findings for future research.

### 4.1. Nature and Extent of Current Evidence

We identified 18 studies published over a period of 20 years. Of the 15 with a specific stated focus on investigating area-level factors, 11 were published in the last 10 years, suggesting a growing recognition of the importance of understanding the impact of area-level factors on employment for people with disability. All the studies were from high-income countries. Further, of the 13 studies from the USA, nine reported research related to vocational rehabilitation or similar programs and thus were restricted to this particular population of people with disability engaged with employment support services.

The studies identified in this review are heterogeneous in terms of methods and data sources, the scale and type of geographic units used for analysis, the disability study population, and the employment outcomes examined. The overall picture is of a disparate collection of studies rather than a body of research that has coalesced around common concepts and approaches.

Using the six domains to organise and report on the examined area-level factors, we identified a concentration of available evidence in the urbanicity domain (15 studies). That so many studies looked at ‘urban versus rural’ (or related categories) may reflect the ready availability of this information in existing data sources. The categories ‘urban’ and ‘rural’ may act as proxies for a constellation of more specific area-level characteristics. For example, restricted access to transportation and low diversity of job types are likely more common in rural areas, and social networks in rural communities may differ from those in urban communities in ways that influence employment outcomes for people with disability.

The current evidence base is uneven in terms of coverage across the six domains, and coverage of specific area-level factors within domains. All eight studies that examined factors in the socioeconomic environment domain included measures related to the local labour market, most frequently unemployment rate, with only four of these studies also examining other socioeconomic factors. The four other domains were represented in four or fewer studies.

Few of the included studies addressed the mechanisms by which area-level factors may operate to influence employment outcomes. This deficit was noted by Galster in relation to the neighbourhood effects literature, that is, relatively little empirical research has investigated the causal pathways via which locational characteristics influence outcomes for individuals [37]. Van Ham and Manley [25] emphasised the crucial role of qualitative data in providing context and elucidating processes that may underpin the associations revealed by quantitative analyses. Only three studies in this scoping review [51,52,56] reported qualitative data, gathered via individual and group interviews, shedding light on the mechanisms by which area-level factors may influence employment outcomes in particular contexts. For example, on the basis of their findings, Hollick [52] suggested that people living in rural areas may experience greater work impairment (absenteeism and presenteeism) due to differences between rural and urban dwellers in job characteristics, such as physical and mental job demands, the amount of autonomy and flexibility afforded to individuals to carry out their work and the level of support offered by employers, as well as access to healthcare services and commuting distances. The authors noted that “Future studies should focus on the effects of mediating factors and their role on the relationship between rurality and work outcomes” [52] (p. 1060).

Several studies discussed how taking area-level factors into account could result in more effective design of policies and delivery of services aimed at improving employment outcomes for people with disability. However, only one used a research design that permitted interactions between area- and service-level factors to be examined by comparing outcomes for standard and best-practice models of service provision in areas with higher and lower local unemployment rates [49]. Similarly, several studies included individual-level factors (e.g., demographic and disability-related variables for individuals), but only one study investigated interactions between area- and individual-level factors [57]. Such interactions deserve more attention to provide evidence concerning how the influence of specific area-level factors may vary for different subgroups of the population with disability.

### 4.2. Limitations

We may not have identified all relevant studies. We aimed to include search terms that would effectively identify studies addressing all three aspects of the research topic: area-level factors, people with disability, and employment outcomes. This was a challenge, as a wide range of terms are used in relation to each aspect. For example, studies focusing on a subgroup of people with disability identified by a particular health condition may have been overlooked if they did not use the terms ‘disability’ or ‘impairment’.

### 4.3. Implications and Directions for Future Research

Van Ham and Manley [25] set out 10 challenges for neighbourhood effects research, all of which we consider pertinent to advancing an understanding of area-level factors associated with employment for people with disability. We highlight four of these 10 challenges as particularly relevant to guide future research. First, there should be greater attention to theorising the mechanisms by which area-level factors may influence employment outcomes, including attention to the spatial scales at which factors and associated mechanisms may operate, and how effects may vary for different subgroups of people with disability. A more extensive body of qualitative studies could assist in generating conceptual models that can inform subsequent quantitative research to investigate specific factors and moderating mechanisms. Second, where a specific area-level factor is found to influence employment outcomes, it is necessary to quantify its contribution to provide an indication of the potential impact interventions that seek to act on that factor may have at a population level. Third, longitudinal research is needed to investigate the temporal dimension to associations between area-level factors and employment outcomes. Where an association between a particular factor and employment outcomes is observed, understanding the role of timing and duration of exposure to that area-level factor is critical for informing effective intervention. Fourth, as there is evidence that people with disability tend to live in more disadvantaged areas [20,64,65], the role of neighbourhood self-selection (the tendency for certain types of households to live in certain geographic areas) in producing observed associations between area-level factors and employment outcomes also needs to be investigated.

Many of the included studies used pre-existing data sources containing administrative data for defined geographic units (e.g., counties). This inevitably places constraints on the area-level factors that can be examined and the spatial scale of geographic units for analysis. Ideally, future research should include purpose-specific spatial data collection and, where possible, seek to create unique neighbourhood units for individuals on the basis of where they live and work. Increasingly, sources of spatial data, linkage methods, and powerful software tools are making it possible to investigate associations between fine-grained environmental exposures and individual-level outcomes [66].

The aim of future research must be to inform policy and intervention design to improve employment outcomes for people with disability. That most studies in this review found associations between urbanicity and employment outcomes suggests that a potentially fruitful focus for future research is to gain a more in-depth and fine-grained understanding regarding what factors underpin urbanicity and how they relate to employment outcomes. Such an understanding could inform interventions designed to address the added disadvantage that may be experienced by people with disability living in non-urban areas.

## 5. Conclusions

Our findings demonstrate that the current knowledge is insufficient to inform the design of interventions that seek to take account of area-level factors to deliver better employment outcomes for people with disability by either modifying environmental factors, or tailoring or targeting interventions with respect to characteristics of the areas in which people live. Concerning future research needed to strengthen the evidence base, we identified the following priorities: theorising the mechanisms by which area-level factors may influence employment outcomes, particularly utilising qualitative research; quantifying the contribution of specific area-level factors to employment outcomes; utilising longitudinal data to explore the role of timing and duration of exposure to area-level factors; investigating the role of neighbourhood self-selection in producing observed associations between area-level factors and employment outcomes; purpose-specific spatial data collection to investigate fine-grained environmental exposures and individual-level outcomes at relevant spatial scales; and interrogating specific area-level factors that underlie associations between urbanicity and employment outcomes for people with disability.

In addition, we recommend that the six domains employed in this scoping review, adapted from the conceptual model developed by Goldfeld and colleagues [45], be used in future research as a structure for identifying and investigating the effects of and interactions between area-level environmental factors in relation to employment outcomes for people with disability.

## Figures and Tables

**Table 1:** Summary of included articles.

No.	Author, Year, Country	Purpose	Study Methods and Geographic Unit	Disability Study Population	Employment Outcome Investigated	Area-Level Domains
1	Becker et al. (2006) USA [46]	To identify predictors of access to supported employment services and rates of competitive employment (efficiency) for people with serious mental illness	Quantitative—survey of 26 mental health agencies; statistical data on local area population, unemployment rate and transportation. Unit: service agency area	Serious mental illness (SMI)	Access to employment services and rates of competitive employment for people with SMI	Physical Socioeconomic Urbanicity
2	Botticello et al. (2012) USA [47]	To assess the role of area-level economic conditions in the likelihood of employment following spinal cord injury	Quantitative—geocoded data from national SCI registry (*n* = 1013); statistical data on area-level measures. Unit: county	Spinal cord injury (SCI)	Employment status of people aged 18–64 with SCI	Socioeconomic Urbanicity
3	Carter et al. (2011) USA [48]	To investigate student, family, school, and community-level factors associated with paid work experiences during high school for youth with severe disabilities	Quantitative—longitudinal survey data on students who received special education services. Unit: community	Youth with autism, intellectual, or multiple disabilities	Paid work experience during high school	Physical Services Urbanicity
4	Cook et al. (2006) USA [49]	To explore effects of local unemployment rates on supported employment programs for people with psychiatric disability	Quantitative—randomised trial (*n* = 1273) within 7 sites using standard or enhanced best practice supported employment (SE) practices; statistical data on area-level unemployment. Unit: county	People with psychiatric disability	Competitive employment, and work for at least 40 h per month	Socioeconomic
5	Edzes et al. (2013) Netherlands [50]	To determine the extent to which a mandatory quota arrangement can create sufficient jobs for the disability target group at local level	Quantitative—spatial analysis comparing quota job opportunities and target population. Unit: municipality	People with disability	Quota jobs available relative to number of people in the disability target group	Governance
6	Gruhl et al. (2012) Canada [51]	To examine access to competitive employment for people with severe mental illness and explore whether place influences access to work	Mixed methods—individual and group interviews with people with severe mental illness and employment service providers (*n* = 46); administrative data on income support beneficiaries from case communities (*n* = 4112). Unit: case community in which employment services provided	People with severe mental illness	Labour force participation	Services Urbanicity
7	Hollick et al. (2020) UK [52]	To examine differences in clinical and patient-reported outcomes, including work, in individuals with axial spondyloarthritis living in rural and urban settings	Mixed methods—data from register for ankylosing spondylitis (*n* = 2390) and interviews with a subset of registry participants (*n* = 30). Unit: urban/rural, not otherwise specified	People with axial spondyloarthritis	Employment status, job type, work missed (absenteeism) or impaired (presenteeism)	Urbanicity
8	Ipsen and Swicegood (2015) USA [53]	To examine rural and urban differences in vocational rehabilitation case mix, delivery practices, and employment outcomes	Quantitative—administrative data from 47 vocational rehabilitation (VR) agencies (*n* = 711,037). Unit: rural–urban commuting area	People with disability	Competitive employment outcome for VR clients	Socioeconomic Urbanicity
9	Ipsen and Swicegood (2017) USA [54]	To explore the viability of vocational rehabilitation (VR) self-employment closures across geography	Quantitative—administrative data from 47 VR agencies (*n* = 711,037). Unit: rural–urban commuting area	People with disability	VR case closure rates to self-employment vs. competitive employment, weekly earnings and hours, and estimated hourly rates	Urbanicity
10	Johnstone et al. (2003) USA [55]	To evaluate differences in demographics, injury severity, and vocational outcomes for persons with traumatic brain injury based on rural vs. urban residency	Quantitative—neuropsychological evaluation and service administrative data for sample of Vocational Rehabilitation (VR) recipients (*n* = 78). Unit: urbanicity, not otherwise specified	People with traumatic brain injury (TBI)	Employment status at VR case closure and VR services received	Urbanicity
11	Landon et al. (2019) USA [56]	To describe vocational rehabilitation professionals’ experiences of the supports and barriers to service provision for people with disability in rural communities	Qualitative—phenomenological analysis of interviews with rural vocational rehabilitation (VR) providers (*n* = 10). Unit: urbanicity, not otherwise specified	People with disability	Perceived success of VR programme	Urbanicity
12	Lustig et al. (2004) USA [57]	To investigate the effect of demographic characteristics and working relationship with a rehabilitation counsellor on employment outcomes for rural and urban consumers with disability	Quantitative—analysis of data from questionnaires provided to rehabilitation service consumers (*n* = 2031). Unit: urban/rural, not otherwise specified	People with disability	Employment status of rehabilitation services consumers	Urbanicity
13	Millet and Sanberg (2003) Sweden [58]	To investigate the influence of individual factors and local area unemployment on the vocational rehabilitation process	Quantitative—data from questionnaires completed by unemployed people registered at vocational rehabilitation programs following period of sick leave (*n* = 143). Unit: urban/rural, not otherwise specified	People aged 18–55 with disability (excluding intellectual disability)	Duration of sick leave and unemployment	Urbanicity Socioeconomic
14	Rabren et al. (2002) USA [59]	To examine variables related to postschool employment status of former special education students	Quantitative—data from survey of students who had experienced a ‘best practice’ transition program. Unit: urban/rural, not otherwise specified	People with disability (predominantly learning or intellectual disability)	Employment status 1 year post-school	Urbanicity
15	Salkever et al. (2018) USA [60]	To explore the impact of client characteristics and a programme initiative on taking up individual placement and support and supported employment by people with severe mental illness	Quantitative—longitudinal analysis of population-based Medicaid cohort data and linked data form other administrative sources. Unit: county	People with severe mental illness (SMI)	Take-up of individual placement and support (IPS) and supported employment (SE)	Service Socioeconomic
16	Sevak et al. (2018) USA [61]	To examine the relationship between employment outcomes and features of the physical, economic, and policy environment for people with disabilities	Quantitative—national survey data linked with state- and county-level environmental variables (*n*= 599,000). Unit: county	People with disability	Employment, hours of work, and earnings	Physical Social Socioeconomic Governance Urbanicity
17	Wong et al. (2020) USA [62]	To compare wages and commute times between workers with and without disability within New York metropolitan region	Quantitative—national survey data. Unit: Intraurban zones	People with/without disability	Wages and commute times	Urbanicity
18	Zhou et al. (2019) Australia [63]	To examine geographic variation in labour force participation rate of people with disability	Quantitative—census data. Unit: Statistical Area Level 2 (SA2)—functional geographic area representing a social and economic community of approx. 10,000 people	People with disability aged 15–64	Labour force participation rate	Social Socioeconomic Urbanicity

**Table 2:** Area-level factors examined by domain.

Domain	Area-Level Factors Examined
**Socioeconomic environment** (1, 2, 4, 8, 13, 15, 16, 18) Covering sociodemographic factors such as population age structure, income, educational attainment, labour force status, and ethnic mix, and features of the local economy such as job availability, industry mix, and economic regeneration and development.	- Unemployment or employment rate (1, 2, 4, 8, 13, 15, 16, 18) - Labour force participation rate (16, 18) - Local labour market characteristics (e.g., share of jobs in blue-collar industries) (16, 18). - Poverty, household income (8, 16, 18) - Education levels (16, 18) - Housing (e.g., % of households in social housing) (18) - Socioeconomic status (2) - Population density (16) - Gender (18) - Ethnicity, language spoken at home (16, 18) - Disability prevalence (18)
**Services** (3, 6, 15, 16) Provision of and access to services, both disability-specific services (e.g., disability employment services) and mainstream services (e.g., banks, shops, government-provided services); measures of service quality and distribution in relation to need.	- Residential proximity to school programming (3) - Employment support services (6) - Supported employment providers (15) - Concentration of physicians (16)
**Physical environment** (1, 3, 16) Including roads, footpaths, parks, housing, presence and accessibility of public transport, and land use patterns.	- Public transport (1, 3, 16) - Transport specifically for people with disability (3)
**Social environment** (16, 18) Including social norms, community social capital, trust, crime, safety, social support networks, civic engagement and neighbourhood attachment.	- Levels of violent crime (16) - Percentage of residents who do voluntary work (18)
**Governance** (5, 16) Covering factors such a policies implemented at local level, leadership, governance structures, partnership structures, and decision-making forums.	- Spatial mismatch between location of job openings for disabled people made available in accordance with a mandatory quota system and the target group of people with disability (5) - Fiscal health of the local government (16)
**Urbanicity** (1–3, 6–14, 16–18) Categorisation such as urban, suburban, rural, metropolitan, and nonmetropolitan based on measures of population density, infrastructure, and/or distance to large cities.	- Metropolitan: yes (>50,000 population)/no (1) - Urban/suburban/rural (2,3) - Urban/rural (6,13) - Urban/rural (defined as settlements of less than 3000 people) (7) - Urban/large rural/small rural/isolated rural (8,9) - Metropolitan/nonmetropolitan (10,12,16) - Rural areas (not further defined) (11) - Rural (county population < 25,000)/urban (county population > 25,000) (14) - Centre/inner ring/suburbs (17) - Major cities/other regions (18)

## Supplementary Materials

The following supporting information can be downloaded at: https://www.mdpi.com/article/10.3390/ijerph19159082/s1, Table S1: Associations between area-level factors and employment for people with disability—specific findings reported, by domain.
