# Supplementary material for: Do Area-Level Environmental Factors Influence Employment for People with Disability? A Scoping Review
Source: Int J Environ Res Public Health. 2022 Jul 26;19(15):9082. doi: 10.3390/ijerph19159082 (PMC9330484; doi:10.3390/ijerph19159082)
Supplement: Supplementary file 1 [file ijerph-19-09082-s001.zip › ijerph-1803401-supplementary.pdf]

Supplementary Table S1: Associations between area-level factors and employment for people with disability – specific findings reported, by domain

Numbers in parentheses refer to the articles as listed below table.

**Socio-economic environment**<br>(1, 2, 4, 8, 13, 15, 16, 18)<br>*covering both socio-demographic factors such as population age structure, income, educational attainment, labour force status, and ethnic mix, and features of the local economy such as job availability, industry mix, and economic regeneration and development.*	- Local unemployment rate negatively associated with employment for people with serious mental illness (1).<br>- Positive association between area socio-economic advantage and employment of people with spinal cord injury; no significant effect of area-level unemployment (2).<br>- Higher local area unemployment associated with less favourable employment outcomes for people with severe mental illness (4).<br>- Economic indicators (area poverty and unemployment rates) are predictors of employment outcomes for VR clients, but account for little variance after controlling for individual demographic, service, and agency variables (8).<br>- Lower area unemployment rate associated with shorter periods of sick leave for people with disability (13).<br>- Negative impact of county unemployment rate on supported employment take-up probability for people with severe mental illness (15).<br>- People with disability living in poor, densely populated areas with high unemployment rates less likely to be employed; more likely to be employed in areas with higher labour force participation rate, larger share of jobs in blue-collar industries, lower proportion of White people and higher proportion of Hispanic people, and larger share of college graduates (16).<br>- Labour force participation rate (LFPR) for people with disability positively associated with % of people with disability who had completed year 12 school, had a post-school qualification, and mainly spoke English at home, LFPR for people without disability, median weekly household income, overall employment rate, and local employment opportunity; but negatively associated with % of people without disability who had a post-school qualification, % of the population with disability, and male-to-female ratio of residents (18)
**Services**<br>(3, 6, 15, 16)<br>*Provision of and access to services, both disability-specific services (e.g., disability employment services) and mainstream services (e.g., banks, shops, government-provided services); measures of service quality and distribution in relation to need.*	- Residential proximity to school programming not associated with employment outcomes for youth with autism, intellectual or multiple disabilities (3).<br>- Employment for people with severe mental illness impacted by distance from, and access to, employment support services (6).<br>- Negative association between driving distance to supported employment provider and supported employment take-up probability (15).<br>- Higher concentrations of physicians associated with lower employment rates and poorer employment characteristics (earnings and weekly hours of work) for people with disability (16).

**Physical environment**<br>(1, 3, 16)<br>*Characteristics of the physical setting including roads, footpaths, parks, housing, presence and accessibility of public transport, and land use patterns.*	- Availability of public transport not predictive of attainment of competitive employment for people with serious mental illness (1).<br>- Positive association between availability of transport for people with disability and paid work for youth with severe disability. Availability of public transport not associated with paid work (3).<br>- Higher level of access to public transportation associated with lower employment rates for people with disability, but with higher earnings for those people with disability who were employed (16).
**Social environment**<br>(16, 18)<br>*Characteristics of the social environment including social norms, community social capital, trust, crime, safety, social support networks, civic engagement and neighbourhood attachment*.	- Higher levels of violent crime associated with lower employment rates for people with disability (16).<br>- Percentage of residents who do voluntary work positively associated with labour force participation rate for people with disability in major cities (18).
**Governance**<br>(5, 16)<br>*Covering area-level implementation of policies, leadership, governance structures, partnership structures, and decision-making forums.*	- Spatial mismatch between location of quota jobs and target group of people with disability; implications considered in relation to decision of national government to decentralise implementation of national policy measures to municipalities (5).<br>- No association between proxy measure for fiscal health of the local government and employment rates for people with disability (16).

**Urbanicity** (1, 2, 3, 6, 7, 8, 9, 10, 11, 12, 13, 14, 16, 17, 18)<br><br>*The designation of an area using categories such as urban, suburban, rural, metropolitan, and non-metropolitan, based on measures of population density, infrastructure, and/or distance to large cities.*	Employment rate:<br>- Community type (rural, suburban, urban) not associated with employment outcomes for adolescents with severe disability (3) or for people with serious mental illness (1).<br>- Likelihood of employment for adults with spinal cord injury was higher in suburban areas compared to urban or rural (people in rural areas at greatest disadvantage) (2).<br>- People with severe mental illness in rural areas experienced higher unemployment than those in urban areas (6).<br>- For people with severe disability, likelihood of employment lower in rural than urban areas; for those with non-severe disability, likelihood of employment higher in rural than urban areas (12).<br>- Odds of having a job one year after leaving school greater for students from urban than from rural settings (14).<br>- Higher population density associated with lower employment rate for people with disability; living in a metropolitan area not significant (16).<br><br>Other employment related outcomes:<br>- Living in rural areas associated with employment in mainly physical/labour-intensive jobs and higher overall work impairment for people with axial spondyloarthritis (7).<br>- Higher educational attainment positively associated with employment in urban but not rural areas (12).<br>- People in urban areas (with lower area-level unemployment) had shorter periods of sick leave than those in rural areas (13).<br>- Living in metropolitan area associated with higher earnings (16).<br>- Wage gap between workers with and without disability greater in central metro and suburban areas than in the inner ring (17).<br>- Commute time for disabled workers greater than for nondisabled workers in the central metro and inner ring areas (17).<br>- For most variables, direction of association with LFPR for people with disability did not vary between major cities and other regions. Home ownership was positively associated in major cities but negatively associated in other regions. Rate of volunteering was positively associated in major cities but not in other regions. Median household income was positively associated and male to female ratio negatively associated in other regions but not in capital cities (18).<br><br>Vocational rehabilitation (VR) outcomes:<br>- Compared to those in rural areas, VR consumers with traumatic brain injury in urban areas receive more maintenance funding, transportation services, and on-the-job training, with a higher percentage employed at VR case closure (10).<br>- Rural locations had higher rates of VR case closures to self-employment and lower rates of closures to employment with supports than urban locations (8).<br>- Closure rates to self-employment increase as geography becomes more rural. Consumers who close to self-employment work fewer hours per week but earn higher hourly wages (9).<br>- VR staff experience of barriers to employment support in rural areas: lack of resources; fear of loss of benefits; fear and mistrust of outsiders; labour market factors (lack of appropriate jobs, informal advertising of jobs); clients' unwillingness to move; lack of

	transportation options; cultural and attitudinal barriers; lack of awareness about VR services among employers and wider community<br>VR staff experiences of facilitators to employment support in rural areas: strong sense of community; support from family networks; supportive employers; community partners (11).

(1) Becker, D.R.; Xie, H.; McHugo, G.J.; Halliday, J.; Martinez, R.A. What predicts supported employment program outcomes? *Community Ment Health J* **2006**, *42(3)*, 303-313.

(2) Botticello, A.L.; Chen, Y.Y.; Tulsky, D.S. Geographic variation in participation for physically disabled adults: the contribution of area economic factors to employment after spinal cord injury. *Soc Sci Med* **2012**, *75(8)*, 1505-1513. https://doi.org/10.1016/j.socscimed.2012.06.010.

(3) Carter, E.W.; Austin, D.; Trainor, A.A. Factors associated with the early work experiences of adolescents with severe disabilities. *Intellect Dev Disabil* **2011**, *49(4)*, 233-247.

(4) Cook, J.A.; Mulkern, V.; Grey, D.D.; Burke-Miller, J.; Blyler, C.R.; Razzano, L.A.; Onken, S.J.; Balser, R.M.; Gold, P.B.; Shafer, M.S. Effects of local unemployment rate on vocational outcomes in a randomized trial of supported employment for individuals with psychiatric disabilities. *J Vocat Rehabil* **2006**, *25(2)*, 71-84.

(5) Edzes, A.J.; Rijnks, R.; van Dijk, J. Spatial implications of using firm level quotas to employ low productive workers. *Tijdschr Econ Soc Geogr* **2013**, *104(5)*, 621-629.

(6) Gruhl, K.R.; Kauppi, C.; Montgomery, P.; James, S. Consideration of the influence of place on access to employment for persons with serious mental illness in northeastern Ontario. *Rural Remote Health* **2012**, *12(3)*, 1-13.

(7) Hollick, R.J.; Stelfox, K.; Dean, L.E.; Shim, J.; Walker-Bone, K.; Macfarlane, G.J. Outcomes and treatment responses, including work productivity, among people with axial spondyloarthritis living in urban and rural areas: a mixed-methods study within a national register. *Ann Rheum Dis* **2020**, *79(8)*, 1055-1062.

(8) Ipsen, C.; Swicegood, G. Rural and urban differences in vocational rehabilitation case mix, delivery practices, and employment outcomes. *Rehabil Res Policy Educ* **2015**, *29(4)*, 349-370.

(9) Ipsen, C.; Swicegood, G. Rural and urban vocational rehabilitation self-employment outcomes. *J Vocat Rehabil* **2017**, *46(1)*, 97-105.

(10) Johnstone, B.; Price, T.; Bounds, T.; Schopp, L.H.; Schootman, M.; Schumate, D. Rural/urban differences in vocational outcomes for state vocational rehabilitation clients with TBI. *NeuroRehabilitation* **2003**, *18(3)*, 197-203.

(11) Landon, T.; Connor, A.; McKnight-Lizotte, M.; Pena, J. Rehabilitation Counseling in Rural Settings: A Phenomenological Study on Barriers and Supports. *J Rehabil* **2019**, *85(2)*.

(12) Lustig, D.C.; Strauser, D.R.; Weems, G.H. Rehabilitation service patterns: A rural/urban comparison of success factors. *J Rehabil* **2004**, *70(3)*, 13.

(13) Millet, P.; Sandberg, K.W. Individual status at the start of rehabilitation: Implications for vocational rehabilitation programs. *Work* **2003**, *20(2)*, 121-129.

(14) Rabren, K.; Dunn, C.; Chambers, D. Predictors of post-high school employment among young adults with disabilities. *Career Dev Except Individ* **2002**, *25(1)*, 25-40.

(15) Salkever, D.; Abrams, M.; Baier, K.; Gibbons, B. Impacting entry into evidence-based supported employment: A population-based empirical analysis of a statewide public mental health program in Maryland. *Adm Policy Ment Health* **2018**, *45(2)*, 328-341.

(16) Sevak, P.; O'Neill, J.; Houtenville, A.; Brucker, D. State and local determinants of employment outcomes among individuals with disabilities. *J Disabil Policy Stud* **2018**, *29(2)*, 119-128.

(17) Wong, S.; McLafferty, S.L.; Planey, A.M.; Preston, V.A. Disability, wages, and commuting in New York. *J Transp Geogr* **2020**, *87*, 102818. https://doi.org/https://doi.org/10.1015j.jtrangeo.2020.102818.

(18) Zhou, Q.S.; Llewellyn, G.; Stancliffe, R.; Fortune, N. Working-age people with disability and labour force participation: Geographic variations in Australia. *Aust J Soc Issues* **2019**, *54(3)*, 323-340. https://doi.org/10.1002/ajs4.75.
